# Cement mantle thickness in total knee arthroplasty more closely associated with tibial bone density than cement viscosity: findings from a randomized controlled trial

**DOI:** 10.1302/2633-1462.74.BJO-2025-0348.R2

**Published:** 2026-04-16

**Authors:** Yoshinori Mikashima, Hitoshi Imamura, Koichiro Yano, Katsunori Ikari, Hiroshi Takagi, Ken Okazaki

**Affiliations:** 1 Oume Knee Surgery Center, Takagi Hospital, Tokyo, Japan; 2 Department of Orthopaedics, Tokyo Women’s Medical University, Tokyo, Japan; 3 Department of Orthopaedics, Tokyo Women’s Medical University Adachi Medical Center, Tokyo, Japan

**Keywords:** Cement viscosity, Cement mantle thickness, Total knee arthroplasty, Tibial bone mineral density, CT, total knee arthroplasty (TKA), bone densities, bone mineral density (BMD), Knees, randomized controlled trial, radiolucent lines, cementless fixation, tibial components, primary TKA, tibial tray

## Abstract

**Aims:**

The aims were to assess the influence of tibial bone mineral density (BMD) and cement viscosity on cement mantle thickness in total knee arthroplasty (TKA), and to determine whether BMD is a more dominant factor than cement viscosity in achieving optimal cement penetration.

**Methods:**

A prospective, randomized controlled trial involving 117 knees from 130 patients undergoing primary TKA was conducted. Patients were allocated to receive either medium-viscosity cement (Group M) or high-viscosity cement (Group H). Cement mantle thickness was measured radiologically at predefined tibial zones using the Modern Knee Society Radiographic Evaluation System. Preoperative tibial BMD was assessed via CT-based quantification. Correlations between tibial BMD and mantle thickness were analyzed, and a cutoff tibial BMD value for achieving ≥ 2.1 mm mantle thickness was determined. This threshold was based on previous findings suggesting that a cement mantle thickness of at least 2.1 mm may help reduce the incidence of radiolucent lines around the tibial component.

**Results:**

No significant differences in mantle thickness were observed between groups at peripheral zones. However, Group H showed significantly greater thickness at central zones (Zone 3 medial (M) (p = 0.001); Zone 3 lateral (L) (p = 0.001); Zone 3 anterior (A) (p=0.001); and Zone 3 posterior (P) ( p = 0.001). Strong negative correlations were found between tibial BMD and mantle thickness in both groups (r = –0.64 to –0.70). The cutoff tibial BMD value to achieve ≥ 2.1 mm mantle thickness was 78.4 HA/cm³. The intra- and inter-rater reliability of the radiological measurements were acceptable (interclass correlation coefficient 0.77 and 0.68, respectively).

**Conclusion:**

Cement penetration beneath the tibial tray is more closely associated with tibial BMD than with cement viscosity. In patients with extremely dense bone, alternative fixation strategies such as cementless fixation or enhanced drilling should be considered.

Cite this article: *Bone Jt Open* 2026;7(4):549–556.

## Introduction

The adoption of cementless total knee arthroplasty (TKA) has risen significantly in recent years.^1^ Several reports have described good clinical outcomes in modern cementless TKA.^2,3^ However, multiple studies in recent years have reported cases of early mechanical loosening in cementless tibial implants.^4,5^ As a result, cement fixation remains the gold standard in TKA.^6^ According to the 2024 American Joint Registry annual report,^7^ approximately 80% of total knee arthroplasties performed in the USA continue to utilize cement fixation.

Adequate cement penetration plays an important role in successful cement fixation. Inadequate penetration can lead to radiolucent lines, which may cause a loss of interlock between the implant and bone.^8-10^ Sasaki et al^9^ reported that a cement mantle of 2.1 mm or more is needed to prevent radiolucent lines.^9^ Furthermore, Schafferler et al^11^ reported that aseptic tibial loosening is associated with the thickness of the cement mantle. Therefore, the depth of cement penetration is crucial for the stability of the implant, potentially leading to a stronger cement-bone interface.^12^

While the use of high viscosity cement (HVC) has decreased compared with a 2017 report,^13^ a recent 2025 statewide registry analysis showed that HVC accounted for 57.9% of all primary TKA cases.^14^ However, comparative evidence regarding its penetration depth compared with that of medium viscosity cement (MVC) in primary TKA remains limited.^13,14^ The primary objective of this prospective study was to determine whether HVC provides better penetration into cancellous bone and a thicker cement mantle than MVC. As a secondary objective, it is hypothesized that the higher tibial BMD is, the thinner cement mantle thickness is, due to reduced porosity of cancellous bone.

## Methods

This prospective, randomized controlled trial was implemented at a single institution following approval from the hospital ethics committee. Written informed consent was obtained from all participants, and the study was registered with the University Hospital Medical Information Network (registration number: UMIN000056520). The investigation period spanned from January to September 2025. The inclusion criteria were: 1) primary TKA for Kellgren-Lawrence (KL) grade 4 medial osteoarthritis and stage 4 osteonecrosis of the medial femoral condyle; and 2) aged between 50 and 89 years. The exclusion criteria were: 1) refusal to participate; 2) diagnosis of rheumatoid arthritis; 3) presence of valgus knees, as their tibial resection levels do not correspond to the CT measurement depths used in this study; 4) knees requiring augmentation; and 5) knees requiring additional bone resection exceeding 11 mm from the lateral tibial joint surface. Eligible patients were randomly assigned to either the MVC or HVC group. Randomized numbers ranging from 1 to 117 were placed into an envelope. Prior to surgery, a sealed envelope was selected by a nursing staff, and the selected number was confirmed. Patients with even numbers were allocated to the MVC group (Group M), and those with odd numbers were allocated to the HVC group (Group H). The baseline demographic details between Group M and Group H are summarized in Table I.

**Table I.** Comparison of baseline demographic details between Group M and Group H.

Variable	Group M (n = 54)	Group H (n = 54)	p-value
Mean age, yrs (SD)	76.5 (7.9)	73.6 (6.9)	0.186
Sex, n	36 females	36 females	
	14 males	16 males	
Mean BMI, kg/m^2^ (SD)	24.8 (3.9)	27.3 (5.3)	0.058
Hip BMD, n %	79.4 (12.8)	82.5 (16.1)	0.455
Medial knee BMD, HA/cm^3^, n %	60.2 (33.3)	73.1 (33.9)	0.188
Lateral knee BMD, HA/cm^3^, n %	44.5 (29.8)	52.8 (30.3)	0.338

BMD, bone mineral density; H, high viscosity cement; HA, hydroxyapatite; M, medium viscosity cement.

Endurance bone cement (DePuy Synthes, USA) was used for cementing in Group M. Palacos bone cement (Heraeus, Germany) was used for cementing in Group H. A pneumatic thigh tourniquet, suction drain tubes, and a foot pump were used for all patients. The preoperative patient demographic details of the two groups are summarized in Table I.

All surgical procedures were performed by a single surgeon (YM) via the medial parapatellar approach with capsulotomy and patellar eversion. Femoral and tibial bone resections were carried out via the measured resection technique. The same posterior stabilized mobile-bearing polyethylene inserts were used in both groups, and patellar resurfacing was performed with cemented anatomically shaped polyethylene patellar components. Furthermore, all tibial components used in this study were of the same design and geometry from a single manufacturer.

After bone resection, two vent holes were created near the lower end of the component in the tibia using a 3.2 mm Steinmann pin.^15,16^ No drilling was performed on the tibial cut surface. The bones were subsequently washed with 1,000 ml of saline solution using the Pulsavac system (Zimmer Biomet, USA). Prior to implant fixation, the CarboJet CO₂ bone preparation system (Kinemed, USA) was used for the removal of debris and dry bone marrow in all cases.

When the bone resections were completed, the cement solution was removed from the refrigerator at 4℃ and stored at a room temperature of 20℃. Two batches of cement were mixed for 1 minute in each mixing cement gun system using a vacuum system. In both groups, 100 mg of amikacin was placed in cement. They were first applied to the tibial, femoral, and patellar implants using a cement gun and then to the bone. The cement was pressed into the bone with a cement gun. Then, impaction was performed. Femoral and patellar cementing was performed via the same method, and a trial insert was inserted. The extruded cement was removed, and the knee was fully extended to further pressurize the cement. After the cement hardened, the knee was flexed, and the excess cement was removed. The cementing techniques used throughout this study were carefully performed for consistency.

**Fig. 1:**
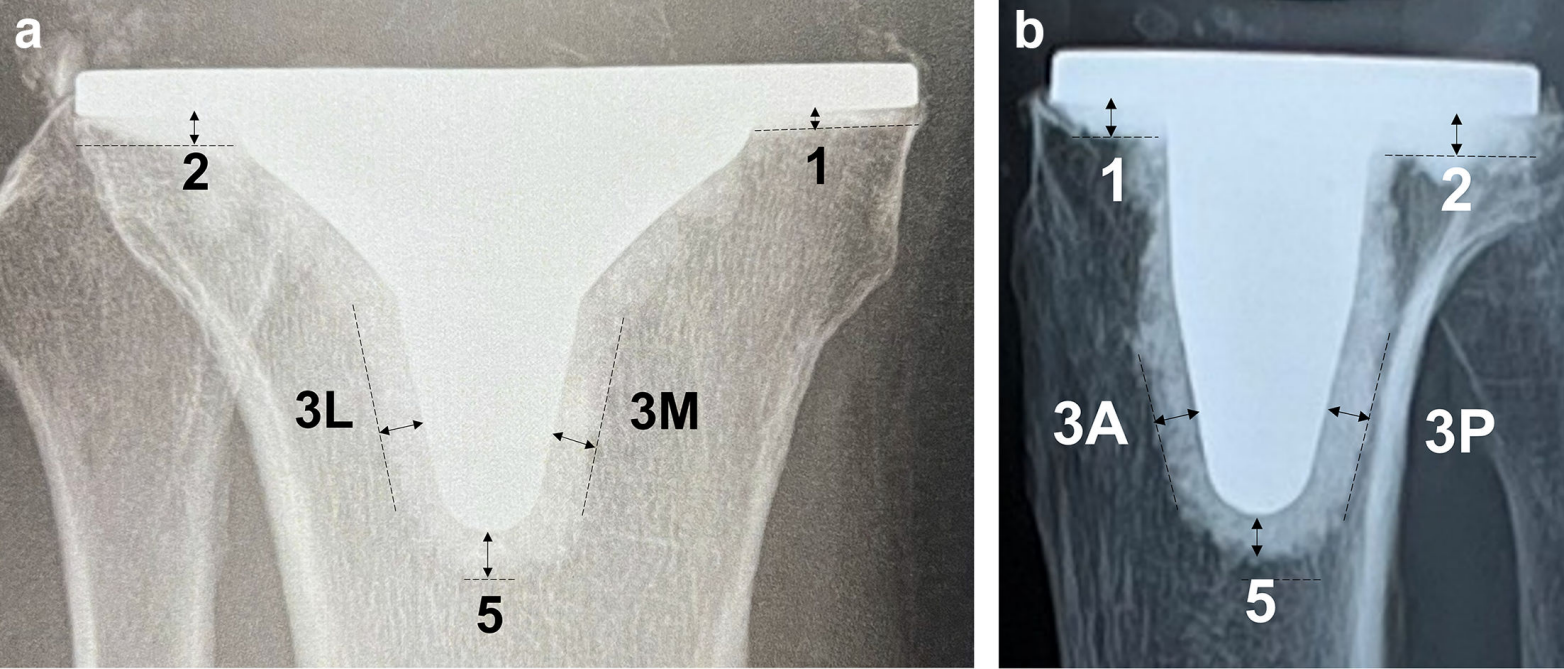
a) Coronal radiographs of implants with zones for documentation of cement mantles. Zone 1: medial baseplate. Zone 2: lateral baseplate. Zone 3: central keel region. Zone 5: the most inferior area of the keel.b) Sagittal radiographs of implants with zones for documentation of cement mantles. Zone 1 of the tibia: anterior baseplate. Zone 2 of the tibia: posterior baseplate. Zone 3 of the tibia: central keel region. Zone 5 of the tibia: the most inferior area of the keel. A, anterior; L, lateral; M, medial; P, posterior.

Clinical radiographs were taken immediately after surgery, as well as at one week, one month, and two months postoperatively, then reviewed and assessed to evaluate the width of the cement mantles. Radiology technicians obtained two serial radiographs; if serial radiographs were unavailable, an additional radiograph was taken to ensure a serial set. Using the Modern Knee Society Radiographic Evaluation System,^17^ the width of the cement mantles was measured by the author (YM). Radiographs identified as the highest quality among serial images obtained during follow-up periods of up to two months were selected for measurement purposes. Three general zones were defined on the anteroposterior (AP) and lateral (L) views of the tibial implant: Zones 1 and 2 to designate the periphery, Zone 3 medial (M), Zone 3L, Zone 3 anterior (A), and Zone 3 posterior (P) to designate the region of the central corn, and Zone 5 to designate the most inferior region of the corn (Figure 1). Cement penetration into the screw holes was not included in the measurements. In this study, zones of the femur were not investigated. The radiological images were enlarged, a digital measurement tool (Synapse; Fuji Medical, Japan) was used to measure the width of the cement mantles, and the results between the two groups were compared.

**Fig. 2:**
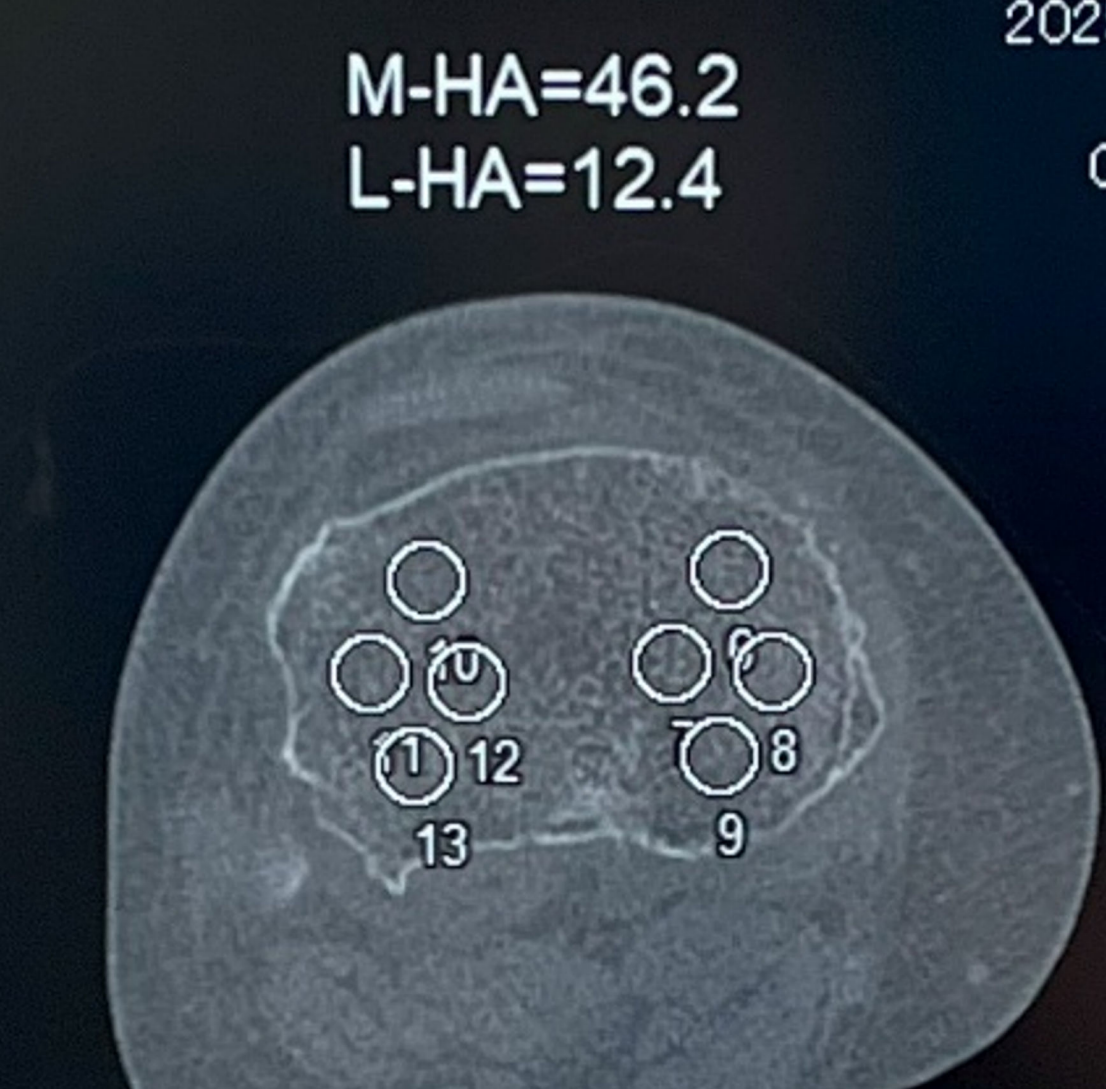
Location of CT measurements for medial and lateral tibial bone mineral density. HA, hydroxyapatite; L, lateral; M, medial.

All patients underwent preoperative assessment of BMD using CT at the proximal tibial resection levels. Preoperative CT scans were evaluated with a CT-based bone density quantification Phantom (mould number B-MAS 200; Kyoto Kagaku, Japan).^18^ This radiology phantom included five cylindrical inserts, each 15 mm in diameter, corresponding to densities of 0, 50, 100, 150, and 200 mg hydroxyapatite (HA)/cm³. During the scan, the phantom was placed beneath the knee scheduled for operation. BMD measurements were taken from a plane parallel to the planned tibial bone cut, specifically 9.0 mm below the lateral surface of the tibia.^18^ From the surgical records, the mean thickness of tibial resection in the most 100 TKAs operated by the same surgeon (YM) was 11.2 mm. Because cartilage thickness has been reported to be 2.1 mm, the tibial BMD was measured 3.0 mm distal to the medial tibial bone surface (medial tibial BMD) and 9.0 mm distal to the lateral tibial bone surface (lateral tibial BMD) on CT, excluding the medial bone sclerosis area (Figure 2).^19^ The relationships between medial and lateral tibial BMD and the width of the cement mantles in Zone 1 and 2 on AP radiographs were investigated. Sasaki et al^9^ reported a cutoff value of 2.1 mm for the cement mantle of the knee where no radiolucent lines appeared. Therefore, the cutoff value of tibial BMD to acquire a width of 2.1 mm or more mantle was also investigated in both groups.

To assess intra- and inter-rater reliability, the widths of cement mantles were measured on 20 randomly chosen radiographs. One examiner took measurements twice, with more than two months between sessions, while a second experienced examiner (HI) also took measurements using the same measurement system. The interclass correlation coefficient (ICC) was calculated to compare the results between groups.

### Statistical analysis

Independent *t*-tests were used to compare the thickness of the cement mantle between the two groups. The correlations between the width of the cement mantle and the preoperative tibial BMD were also analyzed via the Pearson product-moment correlation. To assess the independent effects of tibial BMD, cement viscosity, and other covariates on cement mantle thickness, a multivariable linear regression analysis was conducted. The dependent variable was the radiological cement mantle thickness beneath the tibial tray. Independent variables included age, sex, BMI, tibial BMD, and cement viscosity. Multicollinearity was assessed via variance inflation factors. All statistical tests were two-tailed. Pearson correlation coefficients were interpreted as follows: 0.0 to 0.2, negligible correlation; 0.2 to 0.4, slight correlation; 0.4 to 0.7, strong correlation; and 0.7 to 1.0, very strong correlation. The primary outcomes of this study were the cement mantle thicknesses measured at AP_1 and AP_2. Based on previous literature and pilot data, we assumed a moderate effect size (Cohen’s d = 0.5) for the difference in cement mantle thickness between the two cement viscosity groups. A sample size calculation was performed using a two-tailed *t*-test with an α level of 0.05 and a statistical power of 0.8, resulting in a required sample size of 64 knees per group (128 in total). In addition, a post hoc power analysis assessed the adequacy of the sample size. All analyses were conducted using R Open v. 4.0.2 (R Project for Statistical Computing Austria), and statistical significance was set at p < 0.05.

## Results

**Fig. 3:**
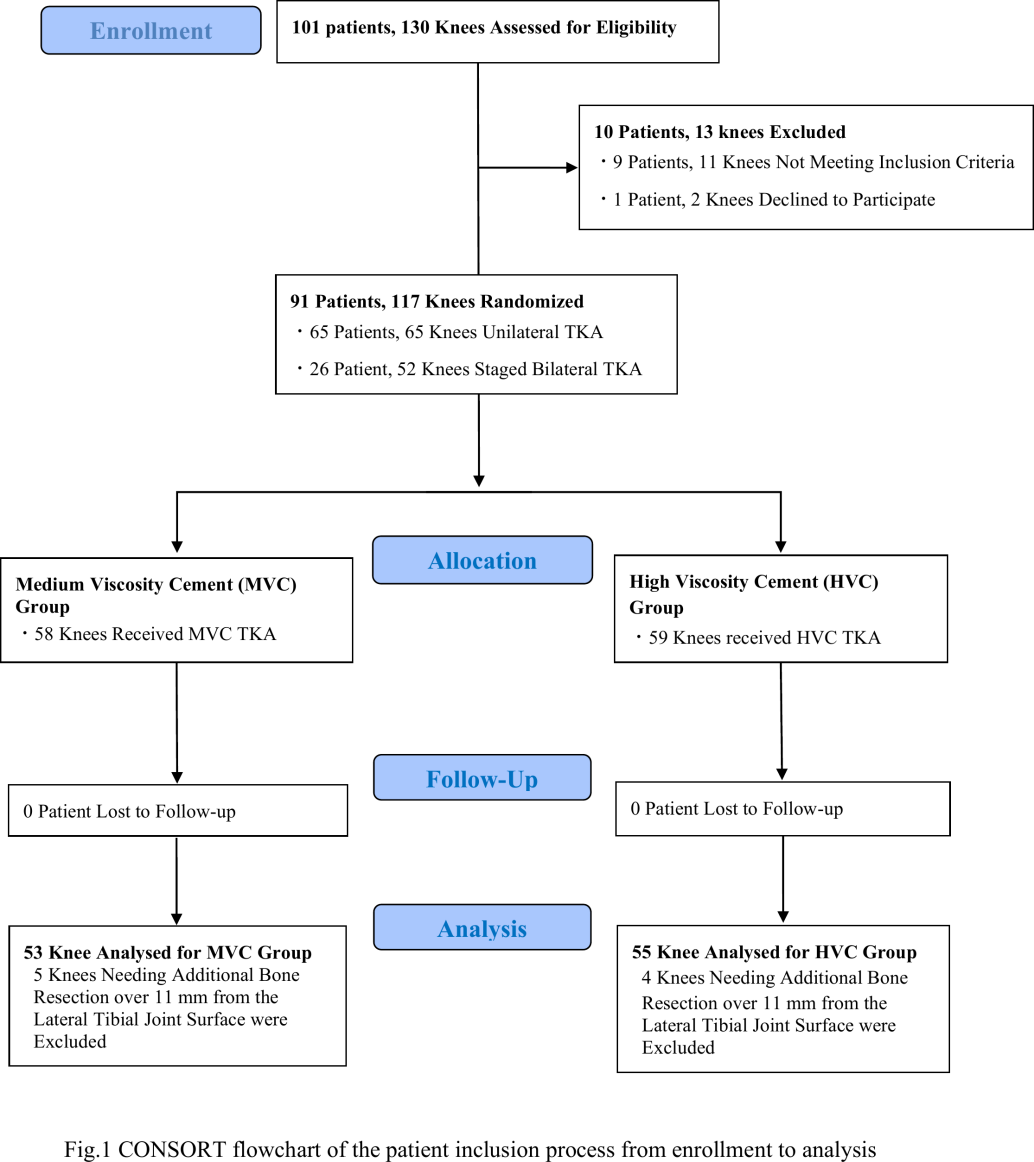
CONSORT flowchart of the patient inclusion process from enrolment to analysis. TKA, total knee arthroplasty.

A total of 58 knees were randomly allocated to Group M, and 59 knees were allocated to Group H (Figure 3). Five knees in Group M and four knees in Group H were excluded from the analysis, because they needed an additional bone resection of over 11 mm from the lateral tibial joint surface.

**Fig. 4:**
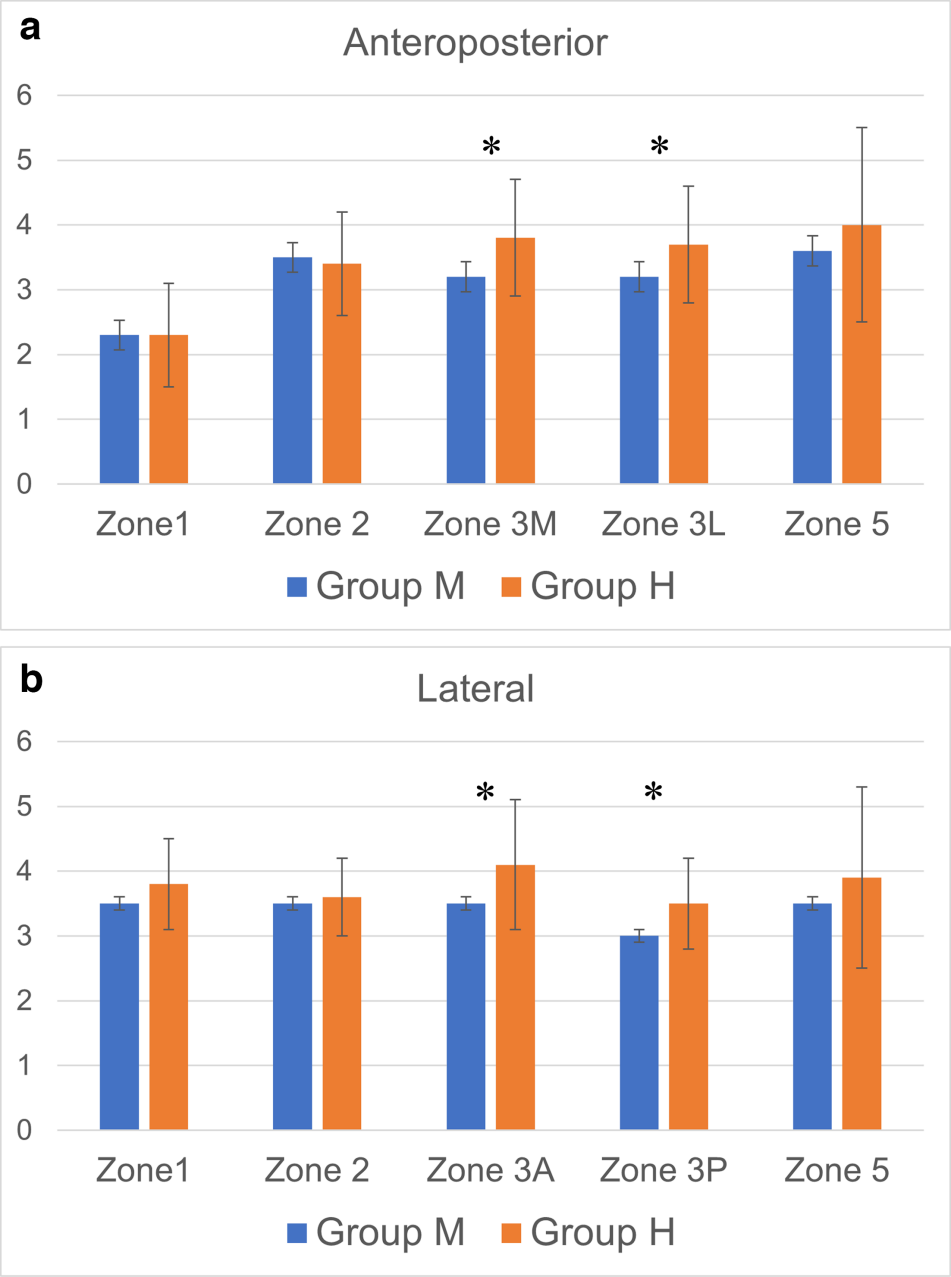
Bar graphs show the mean width of the cement mantle at each zone in the two groups. Error bars represent SD. a) shows those of anteroposterior radiographs, while b) shows those of lateral radiographs. The asterisk indicates a statistically significant p-value. H, high viscosity cement group; M, medium viscosity cement group.

No significant differences were found between groups in demographic characteristics or preoperative tibial BMD (Table I). The mean cement mantle width at each zone is shown in Figure 4. Both groups had similar widths at the proximal tibial zone on AP and lateral views, as well as at Zone 5. However, Group H had significantly thicker cement mantles at Zones 3 M and 3 L on the AP view, and at Zones 3 A and 3 P on the lateral view (Table II).

**Table II.** Comparison of the width of cement mantle between the two groups.

Variable	Group M width, mm	Group H width, mm	p-value
**Mean AP (SD)**			
Zone 1	2.3 (0.9)	2.3 (1.0)	0.956
Zone 2	3.5 (0.8)	3.4 (1.0)	0.502
Zone 3M	3.2 (0.9)	3.8 (0.8)	< 0.001
Zone 3L	3.2 (0.9)	3.7 (1.0)	0.011
Zone 5	3.6 (1.5)	4.0 (1.0)	0.076
**Mean lateral (SD)**			
Zone 1	3.5 (4.0)	3.8 (0.8)	0.119
Zone 2	3.5 (0.6)	3.6 (0.7)	0.341
Zone 3A	3.5 (1.0)	4.1 (1.0)	0.001
Zone 3P	3.0 (0.7)	3.5 (0.8)	0.001
Zone 5	3.5 (1.5)	3.9 (1.1)	0.139

A, anterior; AP, anteroposterior; Group H, high viscosity cement; Group M, medium viscosity cement; L, lateral; M, medial; P, posterior.

**Fig. 5:**
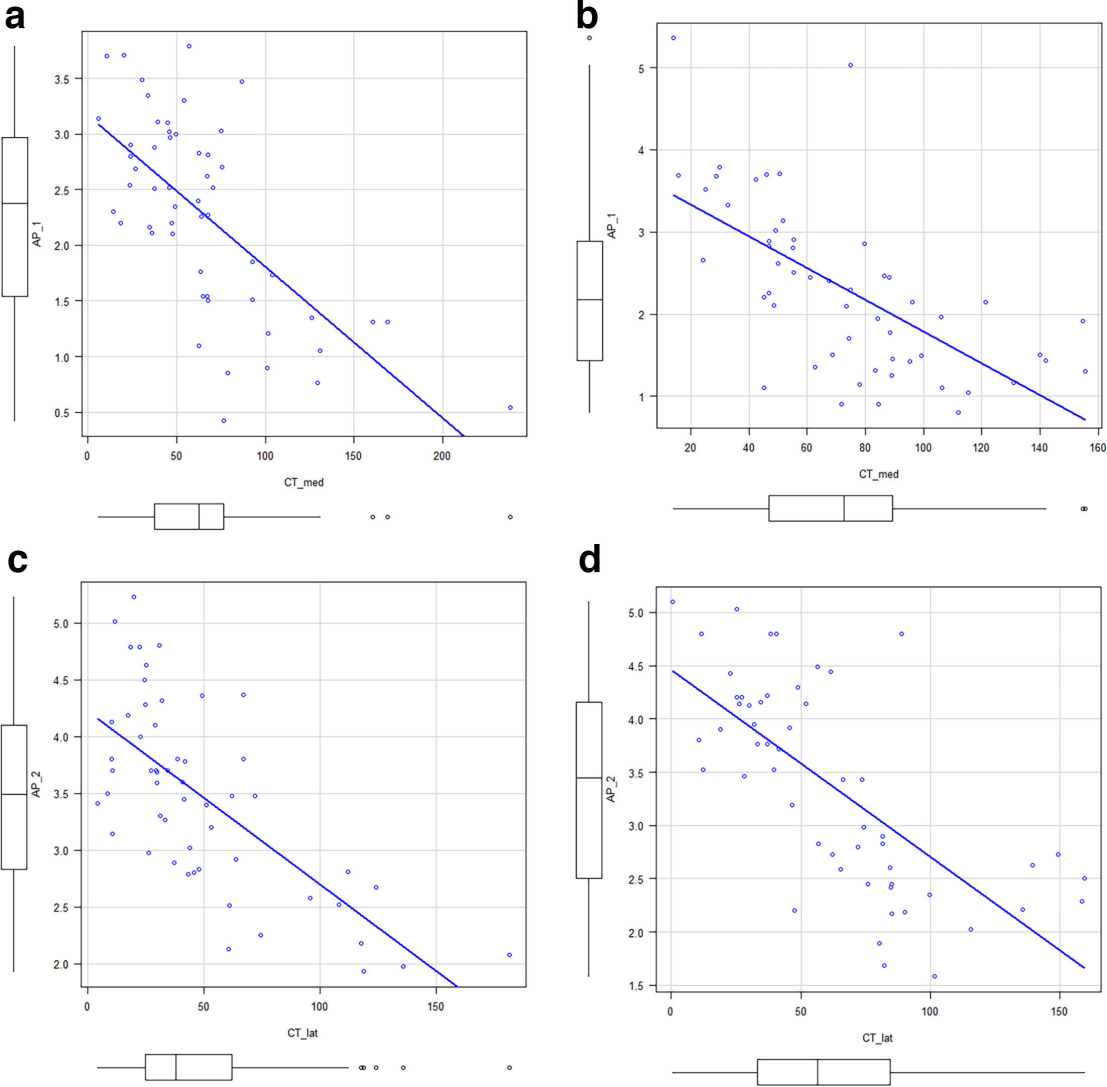
Scatter diagrams indicating the relationships between the width of the cement mantle and the bone mineral density (BMD). The x-axes represent the width of the cement mantle, and the y-axes represent the tibia. a) shows those of Zone 1 of the anteroposterior (AP) radiograph in Group M, and b) shows those of Zone 2 in Group M. Meanwhile, c) shows those of Zone 1 of the lateral (lat) radiograph in Group H, and d) shows those of Zone 2 in Group H. med, medial.

There was a strong correlation between the width of the cement mantle at Zones 1 and 2 on the AP view and each tibial BMD in both groups as described below: Zone 1, Group M: *r* - 0.66 (Figure 5a); Group H: *r* -0.64 (Figure 5b); Zone 2, Group M: r - 0.70 (Figure 5c), and Group H: *r* -0.70 (Figure 5d). The cutoff value of tibial BMD to obtain a cement mantle thickness of 2.1 mm or greater was 76.4 HA/cm^3^. According to the multivariable linear regression analysis, the medial and lateral tibial bone densities were significantly associated with cement mantle thickness (medial: *β* 0.015, p < 0.001, lateral: *β* 0.0185, p < 0.001). Age showed a marginally positive association (*β* 0.026, p = 0.052), whereas cement viscosity, BMI, and sex were not statistically significant (NS). The overall model demonstrated strong explanatory power (adjusted R² 0.544, p < 0.001).

The ICCs of the intra-rater and inter-rater reliability for the radiological measurements were 0.77 and 0.68, respectively.

## Discussion

The most important finding of this study was that cement penetration beneath the tibial tray was not significantly influenced by cement viscosity, but was strongly correlated with the underlying tibial BMD. However, HVC demonstrated significantly greater mantle thickness at the central zones. As tibial BMD increased, cement penetration became shallower, resulting in a thinner mantle regardless of viscosity. This phenomenon reflects the weaker formation of mechanical interlock through 3D penetration into the trabecular structure.^20^ Previous studies have reported that thinner cement mantles may be associated with an increased risk of tibial aseptic loosening, and Sasaki et al^9^ suggested that a mantle thickness of at least 2.1 mm may help prevent radiolucent lines (RLLs).^11,21^ In this study, the proposed cutoff value of tibial BMD to achieve a mantle thickness of 2.1 mm or greater was 78.4 HA/cm³. In knees with a BMD exceeding this threshold, limited cement penetration may occur. Alternative strategies, such as cementless fixation or enhanced drilling may be needed, particularly for patients with high tibial BMD.^15,16,22^

With respect to the cement mantle around the component, HVC resulted in a significantly thicker mantle. This finding is likely attributable to the reduced film squeezing effect inherent to higher viscosity formulations.^12,23^ Luring et al^24^ demonstrated that, compared with baseplate-only cemented models, constructs incorporating both component and baseplate cementing provide superior initial stability and reduced micromotion. Therefore, a thicker cement mantle surrounding the stem may contribute to enhanced mechanical stability. However, the relationship between mantle thickness in this region and long-term clinical outcomes remains insufficiently investigated. Additional studies with extended follow-up are necessary to validate this observation and determine its clinical relevance.

In this study, a uniform cement mantle of approximately 3 mm to 4 mm around the component was obtained in most cases. Lutz et al^25^ reported that the strength of the bone-cement interface is maximized when the cement mantle is uniformly formed at a thickness of 3 mm to 4 mm. There were four potential reasons for these satisfactory results in this study. First, jet washing was thought to have more effectively cleaned the cancellous bone and improved cement penetration.^26^ Second, pressurized carbon dioxide lavage prior to cement fixation could have eliminated debris at the bone-cement interface and may have been effective for increasing cement penetration.^27^ Third, the use of a cement gun prevented the cement from sticking to the gloves and contributed to an even application of cement to the bone.^25^ Finally, in this study, two drill holes with a diameter of 3.2 mm were made around the lower end of the component in the tibia.^16,22^ The bone marrow fluid was able to drain through those vent holes during cement insertion. These techniques were thought to contribute to obtaining a uniform cement mantle.

Recently, the preoperative assessment of tibial BMD using CT has been reported as a useful tool for determining the indications for cementless fixation in TKA.^18,28^ Moreover, a previous study has suggested that CT-derived Hounsfield unit (HU) values are associated with cement penetration depth.^16^ The present study demonstrated that preoperative CT-based tibial BMD is predictive of cement penetration. Knees with high tibial BMD may have risks with cemented fixation. In the near future, bone quality assessment by AI may assist the choice of implant fixation.^29^

This study has several limitations. First, this study did not incorporate postoperative clinical assessments of patients, indicating a need for long-term follow-up investigations to further substantiate these findings. Second, while the sample size was relatively limited, a post hoc power analysis demonstrated adequate statistical power (1–*β* 0.95). Moreover, the final sample size was slightly below the initial target, however, statistically significant differences were observed in the primary outcomes, suggesting that the study was adequately powered to detect clinically meaningful effects. Third, the use of routine CT scans is not universally standard in knee surgery; however, there is a growing body of literature supporting CT-based evaluations of bone quality prior to TKA.^16,18,30^ Fourth, the assessment of bone quality in this study was restricted to the tibia and does not extend to the femoral side or patella. Fifth, the width of the cement mantle was only measured precisely when it was detectable in two serial radiographs. However, two serial radiographs were obtained at regular checkups by radiology technicians. Additionally, while radiological assessments of the cement mantle are subject to some degree of inherent measurement error, efforts were made to minimize this through repeated measurements and reliability testing. Finally, the assessment was performed by the author (YM) without blinding. However, the ICCs of the intra-rater and inter-rater reliability were 0.77 and 0.68, respectively, which were considered acceptable.

In conclusion, this study demonstrated that cement penetration is more closely linked to tibial BMD than to cement viscosity. These findings suggest that an optimal fixation strategy, such as cementless fixation or enhanced drilling, should be considered in cases with extremely dense bone. Long-term follow-up is needed to validate the clinical implications of this study.

**Take home message**

- Cement mantle thickness in total knee arthroplasty is primarily influenced by tibial bone quality.

- Preoperative tibial bone mineral density assessment may guide the fixation strategy, especially in cases with high-density bone.

## Data Availability

The data that support the findings for this study are available to other researchers from the corresponding author upon reasonable request.

## References

[1] Agarwal AR Kuyl E-V Gu A et al. Trend of using cementless total knee arthroplasty: a nationwide analysis from 2015 to 2021 Arthroplasty 2024 6 1 24 10.1186/s42836-024-00241-7 38581037 PMC10998332

[2] Nam D Bhowmik-Stoker M Mahoney OM Dunbar MJ Barrack RL Mid-term performance of the first mass-produced three-dimensional printed cementless tibia in the United States as reported in the American Joint Replacement Registry J Arthroplasty 2023 38 1 85 89 10.1016/j.arth.2022.07.020 35934187

[3] Kaptein BL Toksvig-Larsen S Nelissen R Continued stabilization of a cementless 3D-printed total knee arthroplasty: five-year results of a randomized controlled trial using radiostereometric analysis J Bone Joint Surg Am 2023 105 21 1686 1694 10.2106/JBJS.23.00221 37651549 PMC10609712

[4] Andronic O Yang YH Pabbruwe M Jones CW Yates PJ Early aseptic loosening and inferior patient-reported outcomes of a cementless tibial baseplate in a modern total knee arthroplasty design Bone Joint J 2025 107-B 4 440 448 10.1302/0301-620X.107B4.BJJ-2024-0704.R1 40164184

[5] Gibian JT Zuke WA Hood H et al. Early aseptic tibial loosening is a concern with a modern two-peg cementless total knee arthroplasty design J Arthroplasty 2025 40 3 678 682 10.1016/j.arth.2024.09.023 39307203

[6] Siddiqi A Levine BR Springer BD Highlights of the 2021 American Joint Replacement Registry Annual Report Arthroplast Today 2022 13 205 207 10.1016/j.artd.2022.01.020 35128013 PMC8810304

[7] Ryan SP Stambough JB Huddleston JI Levine BR Highlights of the 2023 American Joint Replacement Registry Annual Report Arthroplast Today 2024 26 101325 10.1016/j.artd.2024.101325 39006856 PMC11239969

[8] Mikashima Y Imamura H Shirakawa Y Yano K Takagi H Okazaki K The vast majority of radiolucent lines disappeared at 3 years follow-up in modern cementless posterior stabilized mobile-bearing total knee arthroplasty Arch Orthop Trauma Surg 2025 145 1 265 10.1007/s00402-025-05880-2 40274614

[9] Sasaki R Nagashima M Tanaka K Takeshima K Relationship between cement penetration and incidence of a radiolucent line around the tibia 2 years after total knee arthroplasty: a retrospective study J ISAKOS 2024 9 4 609 614 10.1016/j.jisako.2024.05.015 38825183

[10] Wautier D Ftaïta S Thienpont E Radiolucent lines around knee arthroplasty components: a narrative review Acta Orthop Belg 2020 86 1 82 94 32490778

[11] Schaffler BC Robin JX Katzman J Arshi A Rozell JC Schwarzkopf R Aseptic tibial loosening is associated with thickness of the cement: a radiographic case-control study J Arthroplasty 2025 40 7 1869 1874 10.1016/j.arth.2024.12.023 39710212

[12] Silverman EJ Landy DC Massel DH Kaimrajh DN Latta LL Robinson RP The effect of viscosity on cement penetration in total knee arthroplasty, an application of the squeeze film effect J Arthroplasty 2014 29 10 2039 2042 10.1016/j.arth.2014.05.010 25007724

[13] Kelly MP Illgen RL Chen AF Nam D Trends in the use of high-viscosity cement in patients undergoing primary total knee arthroplasty in the United States J Arthroplasty 2018 33 11 3460 3464 10.1016/j.arth.2018.07.007 30057268

[14] Cornish ER Zheng H Carpenter C Hallstrom BR Similar revision rates for high- and low-viscosity cement in total knee arthroplasty J Arthroplasty 2025 S0883-5403(25)01216-1 10.1016/j.arth.2025.09.028 41016612

[15] Ahn JH Jeong SH Lee SH The effect of multiple drilling on a sclerotic proximal tibia during total knee arthroplasty Int Orthop 2015 39 6 1077 1083 10.1007/s00264-014-2551-3 25305137

[16] Okuno Y Nagira K Ishida K et al. Comparison of different cementing techniques for cement penetration under tibial component in total knee arthroplasty: a retrospective observational study Knee Surg & Relat Res 2024 36 1 28 10.1186/s43019-024-00232-7 39304941 PMC11414206

[17] Meneghini RM Mont MA Backstein DB Bourne RB Dennis DA Scuderi GR Development of a modern knee society radiographic evaluation system and methodology for total knee arthroplasty J Arthroplasty 2015 30 12 2311 2314 10.1016/j.arth.2015.05.049 26122112

[18] Mikashima Y Imamura H Yano K Ikari K Takagi H Okazaki K Preoperative computer tomography scans can accurately evaluate tibial bone mineral density for selecting bone fixation in total knee arthroplasty J ISAKOS 2025 12 100894 10.1016/j.jisako.2025.100894 40334842

[19] Dório M Hunter DJ Collins JE et al. Association of baseline and change in tibial and femoral cartilage thickness and development of widespread full-thickness cartilage loss in knee osteoarthritis – data from the Osteoarthritis Initiative Osteoarthr Cartil 2020 28 6 811 818 10.1016/j.joca.2020.03.011 32240744

[20] Kodama T The principles of total knee arthroplasty cementing technique – a Japanese perspective In Hansen E Kühn K-D Essentials of cemented knee arthroplasty Springer 2021 511 520 10.1007/978-3-662-63113-3_44

[21] Cox ZC Engstrom SM Shinar AA Polkowski GG Mason JB Martin JR Is cement mantle thickness a primary cause of aseptic tibial loosening following primary total knee arthroplasty? Knee 2023 40 305 312 10.1016/j.knee.2022.12.003 36592499

[22] Refsum AM Nguyen UV Gjertsen J-E et al. Cementing technique for primary knee arthroplasty: a scoping review Acta Orthop 2019 90 6 582 589 10.1080/17453674.2019.1657333 31452416 PMC6844414

[23] Rizzo MG Hall AT Downing JT Robinson RP High-viscosity versus a lower-viscosity cement penetration at dough phase in vivo in primary total knee arthroplasty J Arthroplasty 2021 36 6 1995 1999 10.1016/j.arth.2021.02.010 33707124

[24] Luring C Perlick L Trepte C et al. Micromotion in cemented rotating platform total knee arthroplasty: cemented tibial stem versus hybrid fixation Arch Orthop Trauma Surg 2006 126 1 45 48 10.1007/s00402-005-0082-5 16333631

[25] Lutz MJ Pincus PF Whitehouse SL Halliday BR The effect of cement gun and cement syringe use on the tibial cement mantle in total knee arthroplasty J Arthroplasty 2009 24 3 461 467 10.1016/j.arth.2007.10.028 18534458

[26] Knappe K Bitsch RG Schonhoff M Walker T Renkawitz T Jaeger S Pulsatile lavage systems with high impact pressure and high flow produce cleaner cancellous bone prior to cementation in cemented arthroplasty JCM 2022 11 1 88 10.3390/jcm11010088 35011832 PMC8745275

[27] Sasaki R Nagashima M Otani T et al. Pressurized carbon dioxide lavage reduces the incidence of a radiolucent line around the tibial component two years after total knee arthroplasty J Orthop Surg Res 2022 17 1 349 10.1186/s13018-022-03204-3 35841041 PMC9284780

[28] Lee DH Kwak DS Lee SW Kim YD Cho N Koh IJ Volumetric bone mineral density assessed by dual-energy CT predicts bone strength suitability for cementless total knee arthroplasty Medicina (Kaunas) 2025 61 7 1305 10.3390/medicina61071305 40731934 PMC12300321

[29] Houserman DJ Berend KR Lombardi AV et al. The viability of an artificial intelligence/machine learning prediction model to determine candidates for knee arthroplasty J Arthroplasty 2023 38 10 2075 2080 10.1016/j.arth.2022.04.003 35398523

[30] Choi KY Lee S-W In Y et al. Dual-energy CT-based bone mineral density has practical value for osteoporosis screening around the knee Medicina (Kaunas) 2022 58 8 1085 10.3390/medicina58081085 36013552 PMC9416743

